# Comment on “Predictive factors for distant metastasis in early cutaneous melanoma: a 20-year experience of a Turkish tertiary referral hospital”^؆^

**DOI:** 10.1016/j.abd.2026.501328

**Published:** 2026-03-26

**Authors:** Shyam Sundar Sah, Abhishek Kumbhalwar

**Affiliations:** a Dr. D. Y. Patil Medical College Hospital and Research Centre, Dr. D. Y. Patil Vidyapeeth (Deemed-to-be-University), Pune, MH, India; b Dr. D. Y. Patil Dental College and Hospital, Dr. D. Y. Patil Vidyapeeth (Deemed-to-be-University), Pune, MH, India

Dear Editor,

We read with great interest the study by Ozbagcivan and Kazaz examining predictors of distant metastasis in early-stage cutaneous melanoma across a 20-year Turkish cohort.^1^ The authors should be commended for their long-term follow-up, rigorous histopathological classification, and application of multivariable modeling that incorporated age, Breslow thickness, ulceration, and Lymphovascular Invasion (LVI). The identification of a Breslow thickness cut-off at 2.73 mm offers a potentially practice-informing threshold for risk stratification. However, certain critical interpretive and methodological considerations merit further discussion.

The decision to use a cut-off of 2.73 mm for Breslow thickness based on Receiver Operating Characteristic (ROC) analysis invites scrutiny regarding its generalizability. While the area under the curve of 0.841 suggests good discrimination, the clinical utility of this threshold may be limited in settings where current staging conventions employ simpler strata (≤1 mm, 1–2 mm, etc.) for treatment decisions.^2^ Without external validation or prospective confirmation, this numeric specificity could risk overfitting to a single-center dataset and may not translate effectively into decision-making frameworks such as sentinel lymph node biopsy indication or adjuvant therapy eligibility.

Additionally, while LVI emerged as the strongest histopathological predictor in multivariable models, the study did not evaluate interobserver variability in identifying LVI, which is known to be subject to diagnostic inconsistency.^3^ From a clinical standpoint, LVI reporting often varies by pathology expertise and may be underreported in resource-limited settings.^4^ The absence of standardized immunohistochemical confirmation may limit reproducibility and calls into question the feasibility of integrating LVI as a universal prognostic marker in early melanoma pathways.

The observation that patients over 40-years exhibited significantly higher metastasis rates introduces age as a risk modifier, yet the mechanisms remain speculative. The study would benefit from exploring whether this association reflects age-related biological vulnerability, such as immunosenescence, or diagnostic delay due to reduced surveillance intensity. Clinically, if age is to influence follow-up protocols, further granularity, such as interaction with anatomical site or histologic subtype, would strengthen its translational application.

We commend the authors for producing a comprehensive and geographically distinct melanoma dataset and for identifying population-specific predictors of metastasis. Future multicenter or prospective studies incorporating dermoscopic, molecular, and immunological correlates may enhance predictive accuracy and support integration into risk-adjusted surveillance guidelines.

## ORCID ID

Abhishek Kumbhalwar: 0009-0009-8610-7071

## Generative AI use statement

Grammarly and ChatGPT were tools used solely to assist with language refinement and formatting. All scientific content, interpretations, and critical analyses were developed by the authors, who take full responsibility for the integrity and accuracy of the manuscript.

## Financial support

None declared.

## Authors' contributions

Shyam Sundar Sah: Conceptualization; methodology; writing-original draft; writing-review & editing.

Abhishek Kumbhalwar: Validation; supervision; project administration; writing-original draft; writing-review & editing.

## Research data availability

Does not apply.

## Conflicts of interest

None declared.
